# Characterisation of a functional rat hepatocyte spheroid model

**DOI:** 10.1016/j.tiv.2018.12.014

**Published:** 2019-03

**Authors:** Jonathan A. Kyffin, Parveen Sharma, Joseph Leedale, Helen E. Colley, Craig Murdoch, Amy L. Harding, Pratibha Mistry, Steven D. Webb

**Affiliations:** a Department of Applied Mathematics, Liverpool John Moores University, James Parsons Building, Byrom Street, Liverpool L3 3AF, United Kingdom; b MRC Centre for Drug Safety Science, Department of Molecular and Clinical Pharmacology, Sherrington Building, Ashton Street, University of Liverpool, Liverpool L69 3GE, United Kingdom; c EPSRC Liverpool Centre for Mathematics in Healthcare, Department of Mathematical Sciences, Peach Street, University of Liverpool, Liverpool L69 7ZL, United Kingdom; d School of Clinical Dentistry, Claremont Crescent, University of Sheffield, Sheffield S10 2TA, United Kingdom; e Syngenta Ltd., Jealott's Hill International Research Centre, Bracknell, Berkshire RG42 6EY, United Kingdom

**Keywords:** Liver spheroids, 3D cell culture, Primary rat hepatocytes, Polarisation, Bile canaliculi, Liquid-overlay technique

## Abstract

Many *in vitro* liver cell models, such as 2D systems, that are used to assess the hepatotoxic potential of xenobiotics suffer major limitations arising from a lack of preservation of physiological phenotype and metabolic competence. To circumvent some of these limitations there has been increased focus on producing more representative 3D models. Here we have used a novel approach to construct a size-controllable 3D hepatic spheroid model using freshly isolated primary rat hepatocytes (PRH) utilising the liquid-overlay technique whereby PRH spontaneously self-assemble in to 3D microtissues. This system produces viable spheroids with a compact *in vivo*-like structure for up to 21 days with sustained albumin production for the duration of the culture period. F-actin was seen throughout the spheroid body and P-glycoprotein (P-gp) and multidrug resistance-associated protein 2 (MRP2) transporters had polarised expression on the canalicular membrane of hepatocytes within the spheroids upon formation (day 3). The MRP2 transporter was able to functionally transport 5 μM 5-chloromethylfluorescein diacetate (CMFDA) substrates into these canalicular structures. These PRH spheroids display *in vivo* characteristics including direct cell-cell contacts, cellular polarisation, 3D cellular morphology, and formation of functional secondary structures throughout the spheroid. Such a well-characterised system could be readily exploited for pre-clinical and non-clinical repeat-dose investigations and could make a significant contribution to replace, reduce and refine the use of animals for applied research.

## Highlights

• Initial cell seeding density allows for size-controllability of PRH spheroids.
• PRH spheroids closely resemble *in vivo* morphology and microarchitecture.
• PRH cultured in 3D are an improved system compared to hepatic-derived cell models.
• PRH spheroids may be utilised for long-term repeat-dose investigations
• Cost-effective methodology for the production of primary cell spheroids

## 1 Introduction

The liver is the major organ involved in the metabolism and clearance of numerous xenobiotics. However, detoxification of many of these xenobiotics can contribute to the impairment of liver functionality resulting in injury (Gu and Manautou, 2012). Understanding xenobiotic safety is important to numerous industries such as pharmaceuticals, agrochemicals, industrial chemicals, and consumer products, and therefore robust systems for xenobiotic assessment are required, in particular to support the early research phases of product development (Kyffin et al., 2018). Adverse drug reactions (ADRs) represent a considerable hindrance to the amelioration of novel therapeutics with ~21% of drug attrition attributed to toxicity during the development process (Williams et al., 2013). ADRs are a major cause of liver injury and are responsible for up to 7% of hospital admissions (Pirmohamed et al., 2004). Drug-induced liver injury (DILI), one of the most common forms of ADRs, is the main reason for the withdrawal of drugs from the market (Williams et al., 2013; Eichelbaum et al., 2006; Ingelman-Sundberg, 2008). These reactions are complicated and often require interactions between the multiplex of parenchymal hepatocytes and non-parenchymal cells such as, stellate cells (SCs), Kupffer cells (KCs), liver sinusoidal endothelial cells (LSECs) *etc.* DILI encompasses a vast spectrum of manifestations: the impairment of mitochondrial function, inflammation and lethal effects of immune response, cell death *via* necrosis and apoptosis, and pathologies including microvesicular steatosis and cholestasis (Yuan and Kaplowitz, 2013). *In vitro* liver models that possess the capability to predict potential adverse liver manifestations are greatly valued in the pharmaceutical sector (Andersson, 2017) as well as other industries.

Currently, freshly isolated human hepatocytes cultured in monolayer and sandwich cultures are considered to represent the ‘gold standard’ *in vitro* model for the assessment of hepatotoxic potential of compounds (Gomez-Lechon et al., 2014). However, there are a number of limitations to this model including: the absence of the 3D microenvironment (Soldatow et al., 2013); failure to capture the complexities of multicellularity; inter-donor differences; diminished viability for the study of long-term effects and limited availability to researchers (Godoy et al., 2013). In addition, freshly isolated primary human hepatocytes (PHH) rapidly lose liver-specific functionality and can undergo dedifferentiation within hours of isolation (Gomez-Lechon et al., 2014). As a consequence, the development of alternative *in vitro* 3D liver models has rapidly gained momentum in the field of drug development and hepatotoxicity investigations (Brouwer et al., 2016).

Culturing primary hepatocytes, both human and rat, and hepatic-derived cell lines (C3A, HepG2, Huh7, HepaRG, *etc.*) in a 3D conformation has a profound effect on improving liver-specific functionality, cellular morphology and phenotype, metabolic competence, and toxicological response when compared with 2D cell culture techniques (Messner et al., 2013; Abu-Absi et al., 2002; Sakai et al., 2010; Gaskell et al., 2016; Wrzesinski et al., 2013; Lee et al., 2015; Takahashi et al., 2015). There are multiple methods for generating 3D liver models including hydrogel and scaffold-based technologies (Ramaiahgari et al., 2014; Tibbitt and Anseth, 2009; Anthony et al., 2007; Moscato et al., 2015; Gillette et al., 2011), as well as the production of liver spheroids or hepatospheres (Messner et al., 2013; van Zijl and Mikulits, 2010; Bell et al., 2016; Li et al., 2008). There are a number of 3D spheroid culture protocols for monocultures of both hepatocytes and cell lines as well as cultures that include secondary or multiple cell types, all of which vary according to individual practices (Messner et al., 2013; Ramaiahgari et al., 2014; Phung et al., 2011; Gomez-Lechon et al., 1998; Kostadinova et al., 2013). Spheroids have the potential to be used in both long-term and repeat-dose investigations, with many platforms allowing for high-throughput analysis for pre-clinical and non-clinical investigations. The improved liver-specific functionality of spheroids has been assessed *via* numerous end-point analyses such as, albumin and urea production; and the up-regulation of key cell adhesion molecules (integrin 3, cadherin 1, connexin 32), transcription factors (HNF4α), and the metabolising enzyme cytochrome P450 7A1 (CYP7A1) (Sakai et al., 2010).

Hepatic-derived cell lines such as HepG2 and C3A cells possess a number of attractive characteristics such as: nuclear factor erythroid 2-related factor 2 (Nrf2) expression (Hagiya et al., 2008); unlimited growth and availability; and the absence of inter-donor variability ensuring reproducible results (Castell et al., 2006). These cell lines are easily maintained and are uncomplicated to culture (Jennen et al., 2010). For these reasons, researchers have carried out numerous primary toxicological and pharmacological studies using these cells cultured as spheroids. However, some of the main limitations that remain with spheroid models that utilise hepatic-derived cell lines are their limited metabolic capacity in direct comparison with primary hepatocytes (Guguen-Guillouzo and Guillouzo, 2010), and the formation of necrotic regions throughout the microtissues due to the proliferative nature of the cells.

One of the main advantages that primary hepatocytes have over hepatic cell lines is that they do not proliferate and thus, the size of the resulting spheroids remains relatively constant over time. Furthermore, for an *in vitro* model that attempts to reproduce the microenvironment of the healthy *in vivo* liver, the formation of necrosis is highly unrepresentative. The stability of primary hepatocyte spheroid sizes over the duration of the culture period may allow for the sufficient diffusion of oxygen and other key nutrients throughout the entirety of the microtissue, and this may arrest the formation of necrosis.

One of the inherent characteristics of hepatocytes *in vivo* is their ability to polarise, both structurally and functionally. Key transporters are expressed on either the apical (canalicular) or the basolateral (sinusoidal) membrane of the hepatocytes (Esteller, 2008). Along with this transporter localisation, bile canaliculi form between adjacent hepatocytes affirming cellular polarisation (Müsch, 2014; Gissen and Arias, 2015). The formation of bile canalicular structures has been demonstrated with primary rat hepatocytes previously indicating a morphology close to that of *in vivo* (Abu-Absi et al., 2002). As well as the formation of bile canalicular-like structures, cells within these rat hepatocyte spheroids have exhibited polarisation, assessed by the staining of apical HA4 and basolateral HA321 membrane-bound proteins (Abu-Absi et al., 2002) and the use of dipeptidyl peptidase IV (DPP IV) as an apical membrane marker (Wang et al., 2008).

To date, 3D liver spheroids have been shown to be an improved *in vitro* model when compared with conventional 2D cultures as 3D models exhibit enhanced liver-specific functionality, increased culture longevity, and improved sensitivity to hepatotoxicants (Gaskell et al., 2016; Riccalton-Banks et al., 2003; Thomas et al., 2005). Previous research with PRH spheroids has demonstrated the formation of bile canaliculi-like structures and some of their functional properties (Abu-Absi et al., 2002). However, there are other key attributes and functions that need to be comprehensively evaluated in PRH spheroids such as cellular morphology and polarisation, and the formation of secondary structures and their subsequent functionality. Size controllability of spheroids has previously been identified as a limitation of rocked and spinner flask cultures. However, for other platforms that overcome this limitation, such as the commercially available InSphero platform, the high cost of utilising this system becomes a major limiting factor. In this study, we have developed a cost-effective 3D liver spheroid model by culturing PRH utilising the liquid-overlay technique (LOT). This *in vitro* system has been well characterised by numerous end-points including structural and functional parameters. However, much of the previously reported investigations have utilised spinner flasks and rocked cultures and therefore the size-controllability of these *in vitro* tools is a limitation. We demonstrate a novel implementation of the LOT with PRH to produce size controlled spheroids that display many of the *in vivo*-like characteristics required for xenobiotic safety assessments.

## 2 Materials and methods

### 2.1 Materials

All materials were purchased from Sigma-Aldrich, UK, unless otherwise stated.

### 2.2 Production of liquid-overlay plates

Spheroids were produced using the liquid-overlay technique as previously described (Yuhas et al., 1977). A solution of 1.5% agarose (high gelling temperature) in serum-free Williams' medium E was produced. The solution was autoclaved and subsequently 100 μl of the solution was added to each well of a flat-bottomed 96-well plate. Plates were left for 30 min for agarose to fully solidify. Lids were placed back onto 96-well plates and the plates were inverted and stored at 4 °C for 2 weeks to allow for appropriate hydration prior to cell seeding.

### 2.3 Primary rat hepatocyte isolation and culture

Hepatocytes were isolated from the whole liver of an adult male Wistar rat, weighing between 175 and 200 g, by a modified two-step collagenase perfusion technique described by Seglen (Seglen, 1976). Briefly, the rat liver was perfused through the portal vein with Ca^2+^/Mg^2+^-free HBSS containing 10 mM glucose, 10 mM HEPES, and 0.3 mM EDTA followed by HBSS containing 0.05% collagenase type IV. The cell suspension was spun down at 50 *g* for 2 min. After three washes, hepatocytes were resuspended in Williams' medium E, supplemented with 10% FBS, 10,000 units/ml penicillin, 10 mg/ml streptomycin, 2 mM l-Glutamine, 10 μg/ml insulin, 5.5 μg/ml transferrin, 6.7 ng/ml selenium and 100 nM dexamethasone under standard culture conditions, (this will hereafter be referred to as supplemented media). Cell viability was determined using the trypan blue exclusion method where 40 μl of the cell suspension was transferred to a 0.5 ml microcentrifuge tube with 10 μl of trypan blue stain. 10 μl of the resultant cell suspension was loaded onto a haemocytometer and cells were counted (both total and viable). Isolated cell populations with >85% viability were used for subsequent spheroid cultures.

### 2.4 Spheroid formation

PRH were seeded at 2000, 3000, 4000 and 5000 cells per well in 100 μl of supplemented media. Plates were briefly pulse spun in a plate spinner at 100 x *g* for 90 s and incubated for 72 h to allow spheroid formation. After this time, media was changed twice weekly where 50 μl was removed and replenished with another 50 μl of supplemented media. Spheroids were cultured for up to 21 days. Spheroid diameter was analysed *via* light microscopy utilising a phase-contract microscope (ECLIPSE TS100/100-F, Nikon). Images were taken at 4× magnification using a digital camera head (DS-Vi1, Nikon) and a stand-alone controller and display unit (DS-L3, Nikon). The maximum spheroid diameter was measured from these images (*n* = 10).

### 2.5 Histological analysis

Spheroids were washed in PBS, fixed for 1 h in 4% paraformaldehyde (PFA) and embedded in 2% agarose (low EEO) in 4% PFA before being subjected to routine histological processing and finally paraffin wax embedded. 5 μm sections were cut using a Leica RM2235 microtome (Leica microsystems) and stained with Haematoxylin and Eosin (H&E) or subject to immunohistochemical (IHC) analysis.

### 2.6 Immunohistochemical analysis

Sections were dewaxed, rehydrated through a series of alcohol dilutions, and endogenous peroxidase neutralised with 3% hydrogen peroxide in methanol for 20 min. Antigen retrieval was achieved by using 0.01 M Tri sodium citrate buffer (pH 6) at high temperature. After blocking with normal goat serum for 20 min at room temperature, sections were incubated with primary antibodies at a 1:50 dilution for cleaved-caspase 3 and a 1:500 dilution for Vimentin (1:50 dilution Ki67, Abcam, Cambridge, UK (not shown)) for 1 h at room temperature. Secondary antibody and avidin-biotin complex (ABC) provided with Vectastain Elite ABC kit (Vector labs, Peterborough, United Kingdom) were used in accordance with the manufacturer's instructions. Finally, 3¢-diaminobenzidine tetrahydrochloride (DAB; Vector labs) was used to visualise peroxidase activity and the sections were counterstained with haematoxylin, dehydrated, and mounted in DPX. Light microscope images were taken using an Olympus BX51 microscope and Colour view IIIu camera with associated Cell^D software (Olympus soft imaging solutions, GmbH, Münster, Germany).

### 2.7 Analysis of cellular ultrastructure *via* transition electron microscopy (TEM)

Spheroids were fixed in fresh 2.5%–3% glutaraldehyde in 0.1 M phosphate buffer, overnight at 4 °C. Spheroids were then washed in 0.1 M phosphate buffer twice with 30-min intervals at 4 °C. Secondary fixation was carried out in 2% OsO_4_ for 2 h at room temperature and pressure and then washed in 0.1 M phosphate buffer. Dehydration occurred through a graded ethanol series. Infiltration was accomplished by a 50/50 mixture of Propylene oxide/Araldite resin. Specimens were embedded in Araldite Epon for 6–8 h at RTP and then placed in fresh Araldite Epon for 48–72 h at 60 °C. Ultrathin sections (~70–90 nm) sectioned by Reichert Ultracut E ultramicrotome and stained by 3% aq. uranyl acetate and Reynold's lead citrate for 5 min. Sections were examined using a FEI Tecnai Transmission Electron Microscope at an accelerating voltage of 80 Kv. Images were taken using a Gatan digital camera.

### 2.8 Immunofluorescence analysis

Spheroids were transferred to flat-bottomed 96-well plates, washed three times in PBS and subsequently fixed in 4% PFA for 1 h at 4 °C. Spheroids were incubated in a permeabilization buffer (0.5% Triton X-100 in Tris-buffered Saline with 0.5% Tween20 (TBST)) overnight at 4 °C and then blocking buffer (0.1% Triton X-100, 3% BSA in TBST) for 2 h at room temperature. Primary antibodies (P-gp-Abcam and MRP2-Abcam) were diluted 1:50 in 0.1% Triton X-100, 1% BSA in TBST and spheroids were incubated with primary antibodies overnight at 4 °C. Spheroids were then washed 3 times for 1 h in 0.1% Triton X-100 in TBST and then incubated with secondary Alexa Fluor 568 donkey anti-mouse and Alexa Fluor 568 donkey anti-rabbit (Thermo Fisher Scientific) diluted to 1:1000, with Hoechst diluted to 1:5000 and Phalloidin 680 diluted to 1:250 in 0.1% Triton X-100, 1% BSA in TBST overnight at 4 °C. Spheroids were then washed 2 times for 1 h in 0.1% Triton X-100 in TBST and mounted onto a microscope slide with ProLong Gold antifade mountant (Thermo Fisher Scientific for P-gp and MRP2 respectively). Maximum intensity projection images were taken using a Zeiss Axio Observer microscope with Apotome platform using 10× and 40× oil objective.

### 2.9 Analysis of transporter functionality

Spheroids were incubated with 5 μM 5-chloromethylfluorescein diacetate (CMFDA; Thermo Fisher Scientific) a non-fluorescent cell tracker which has been shown to be cell permeable until it is converted by cytosolic esterases into glutathione-methylfluorescein (GSMF), an auto-fluorescent substrate that is transported by MRP2 (Förster et al., 2008). Spheroids were incubated with CMFDA for 2 h at 37 °C. Spheroids were subsequently washed three times in PBS and prepared for IF as previously described.

### 2.10 Urea and albumin quantification

Albumin and urea synthesis were quantified in spheroid media supernatant samples using Rat Albumin ELISA Kit (Abcam) and Urea Assay kit (Abcam) following the manufacturer's protocol. Supernatant samples were collected 4 days after media changes over the 21-day culture period. Data was normalised to account for cell number variation.

### 2.11 Statistical analysis

Quantitative data are presented as descriptive [mean ± standard error and inferential statistics (*p* values). Statistical significance was evaluated *via* One-way ANOVA. Significance was set at *p* < .05. Data presented is representative of at least 3 independent experiments (*n* = 3) in triplicate. Graphs were produced and analysed using GraphPad Prism 5 (GraphPad software, San Diego, CA, USA).

## 3 Results

### 3.1 Optimization of spheroid production

**Fig. 1:**
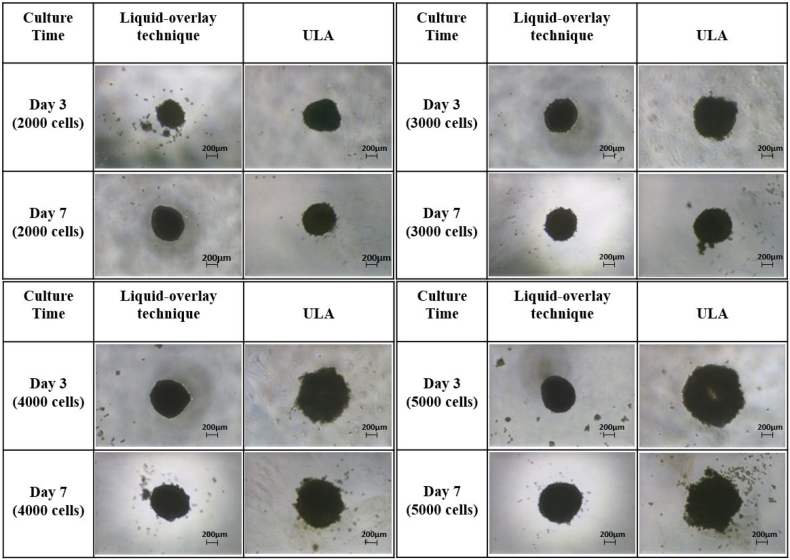
Formation and morphology of spheroid produced using the liquid-overlay technique (LOT) or ULA plates for different initial seeding cell numbers. Fig. 1 shows phase-contrast images of spheroids produced using 2000, 3000, 4000 and 5000 PRH at day 3 and day 7. Scale bar = 200 μm.

In the initial stages of experimentation, we compared spheroid formation utilising two different techniques: the LOT and ultra-low adhesion (ULA) plates. It was noted that spheroids grown in the ULA plates were more irregular, less spherical, not as compact, and had a less well-defined membrane around the periphery of the spheroids when compared to spheroids cultured using the LOT (Fig. 1). All further experimentation was carried out on spheroids that had been produced using the LOT.

### 3.2 Effect of initial cell seeding number on spheroid size and morphology

**Fig. 2:**
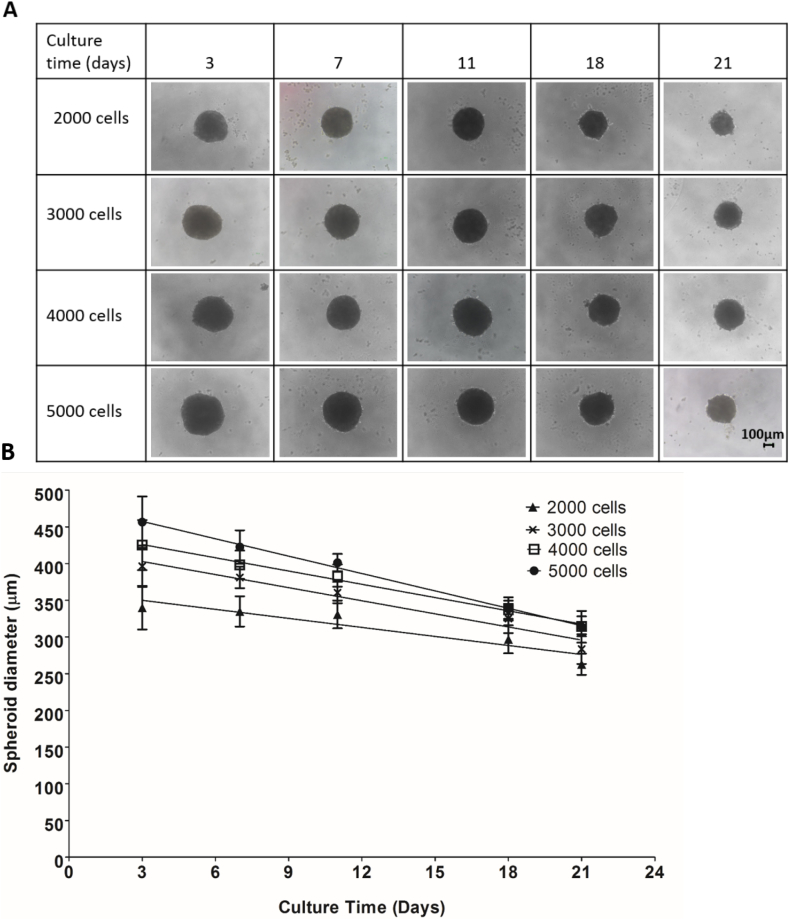
Effect of initial cell seeding density on spheroid size and morphology. Spheroids were produced from seeding 2000, 3000, 4000 and 5000 *PRH* per well in 100 μl complete Williams' medium E and cultured for up to 21 days. (A) phase-contrast images of spheroids taken at time points (3, 7, 11, 18, and 21 days). Scale bar = 100 μm. (B) compaction of spheroids/reduction of spheroid diameter over 21-day culture period. Data are represented as mean ± SD (n = 10 in triplicate). Straight lines represent linear regression analysis using Graphpad prism. R^2^ values were 0.768, 0.9569, 0.9928 and 0.9957 for 2000-, 3000–4000- and 5000-cell spheroids respectively.

To determine how the number of PRH initially seeded affected spheroid morphology and size, spheroids were produced by seeding 2000, 3000, 4000 and 5000 cells and monitored over 21 days. The initial seeding density resulted in spheroids of varying sizes, with all cells initially seeded aggregating to form a single uniform spheroid within 3 days. Spheroids produced from the seeding of 2000 cells reduced in diameter over the 21-day culture period from 339.92 ± 29.78 μm to 262.88 ± 14.43 μm. 3000-cell spheroids reduced in diameter over the culture period from 396.41 ± 28.29 μm to 283.47 ± 20.23 μm, whilst 4000- and 5000-cell spheroids reduced in diameter of the culture period from 425.10 ± 34.08 μm to 314.50 ± 13.64 μm and 456.48 ± 34.92 μm to 313.83 ± 21.55 μm, respectively. All spheroids remained relatively uniform in shape over the duration of the culture period (see Fig. 2).

**Fig. 3:**
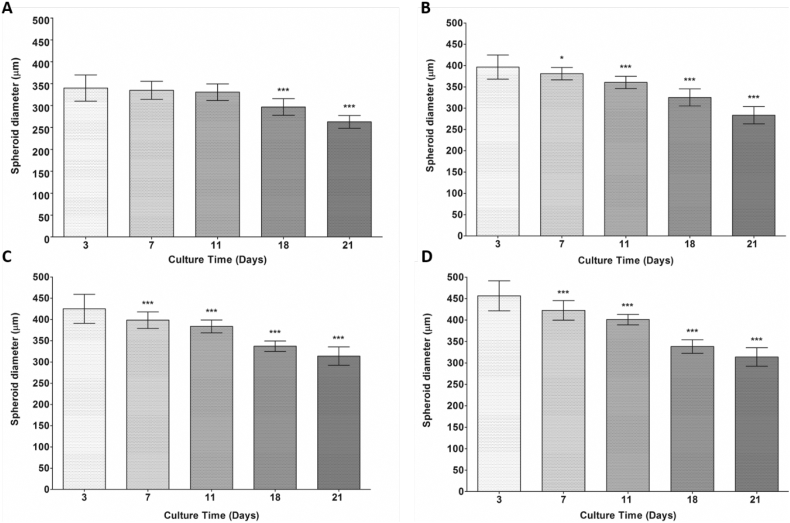
Statistical analysis of spheroid compaction. Statistical analysis of spheroid compaction over culture time. There was a time dependent decrease in spheroid diameter up to 21 days in culture. Fig. 3 (A) describes the decrease in spheroid diameter for 2000-cell spheroids, (B) for 3000-cell spheroids, (C) for 4000-cell spheroids and (D) for 5000-cell spheroids. For 2000-cell spheroids, days 18, and 21 were considered statistically significantly smaller when compared to day 3. All other spheroid diameters for 3000, 4000 and 5000-cell spheroids were considered statistically significantly smaller compared to day 3 spheroids, by One-way ANOVA *P value < .05 and ***P value < .001. Data are represented as mean ± SD.

### 3.3 Histological analysis of spheroids

**Fig. 4:**
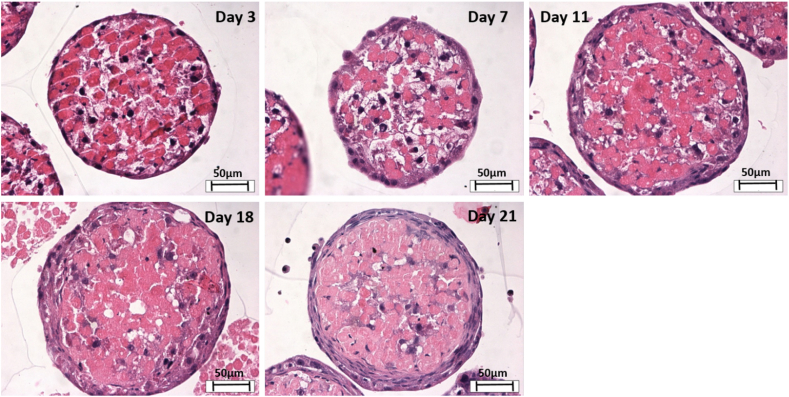
Spheroid internal structure and morphology. Spheroids were created on liquid-overlay plates from 5000 cells per well in 100 μl of complete medium and fixed at day 3,7,11,18, and 21 of culture. Samples were subsequently paraffin embedded, sectioned and stained with H&E. Images represent mid-sections through the spheroids at 40× magnification. Scale bar = 50 μm.

**Fig. 5:**
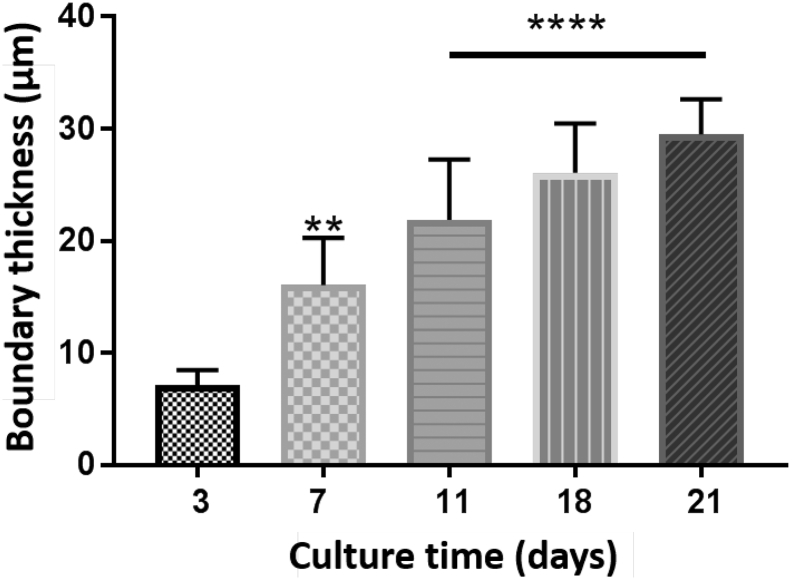
Statistical analysis of peripheral cell thickness. Graph describing spheroid cell perimeter thickness. ImageJ was used to quantify cell-perimeter thickness overtime. There was a time dependent increase in cell perimeter thickness up to 21 days in culture. For the calculation, six areas along the perimeter were measured per spheroid. All spheroid cell perimeters were considered statistically significantly thicker compared to day 3 spheroids by One-way ANOVA **P value < .0022 and ****P value < .0001.

Haematoxylin and Eosin (H&E) staining was performed on spheroids with an initial cell seeding number of 5000 cells over a 21-day culture period to assess cell morphology and cellular arrangement (Fig. 4). H&E staining revealed a compact internal cellular arrangement with a defined outer perimeter of cells. However, the outer perimeter of cells increased in thickness over the duration of the culture period (see Fig. 5 (statistical analysis)). It was also noted that over the duration of the culture period, staining consistent with the formation of central areas of necrosis was not observed.

### 3.4 Nuclear staining of spheroid sections

**Fig. 6:**
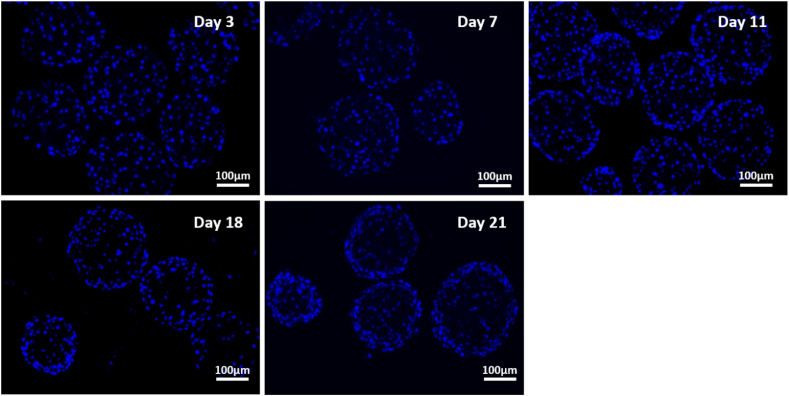
Cellular morphology and nuclear distribution within spheroids. Spheroids were created on liquid-overlay plates with 5000 cells per well in 100 μl of complete media and fixed at day 3, 7, 11, 18, and 21 of culture. Samples were subsequently paraffin embedded, sectioned and stained with Hoechst. Images represent mid-sections through the spheroids at 20× magnification. Scale bar = 100 μm.

The internal structure of the spheroids was analysed using immunofluorescent (IF) microscopy. 5 μm-sections from 5000-cell spheroids were stained with Hoechst over 21 days of culture. Nuclear staining was visualised to assess cellular arrangement within the spheroid (Fig. 6). Sections from the same blocks as in Fig. 4 were analysed for direct comparison. H&E stained sections displayed some areas of intense eosinophilic staining and subsequently made nuclear staining difficult to determine. IF analysis was required to better visualise nuclear staining and cellular arrangement within the spheroid over the culture period.

### 3.5 Immunohistochemical analysis

**Fig. 7:**
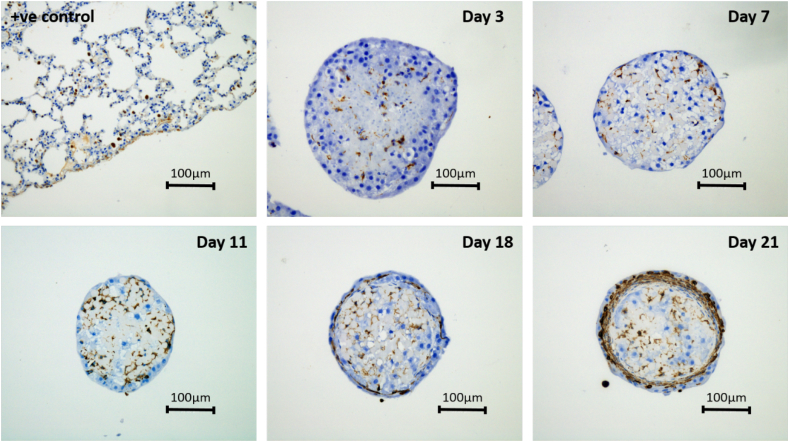
Vimentin staining for boundary cell characteristic analysis. Spheroids were created on liquid-overlay plates from 5000 cells per well and fixed at day 3, 7, 11, 18 and 21 of culture, paraffin embedded, sectioned and stained with Vimentin. Slides were counterstained with haematoxylin (blue) to stain for cell nuclei. Intense brown staining shows the presence of mesothelial cells. Images represent mid-sections through the spheroids at 20× magnification. Scale bars = 100 μm. (For interpretation of the references to colour in this figure legend, the reader is referred to the web version of this article.)

The internal structure of the spheroids was analysed using (IHC) staining to assess if there was a secondary cell type present from the initial hepatocyte isolation procedure that may have been forming the encapsulated boundary. Vimentin staining was performed on 5000-cell spheroids over 21 days of culture. Vimentin has been shown to positively stain for mesothelial and fibroblast cells and as such this stain was chosen to determine whether or not the cells on the boundary of the spheroids where different in phenotype when compared to the isolated hepatocytes (Fig. 7). IHC positive control slides were produced from rat liver sections which stained positively for mesothelial/fibroblast cells throughout the tissue, but more specifically at the boundary of the samples. Positive staining could be seen over the 21-day culture period for mesothelial/fibroblast cells, however, this staining was increasingly constrained to the peripheral cells within the spheroids as the culture time increased most likely indicating the presence of the mesothelial lining due to the locality of the staining. Ki67 staining was also carried out to determine whether or not the cells located at the spheroid periphery were proliferative. Ki67 staining was predominantly negative for all time points of the analysis suggesting that the secondary cell type is much more likely to be mesothelial in nature.

**Fig. 8:**
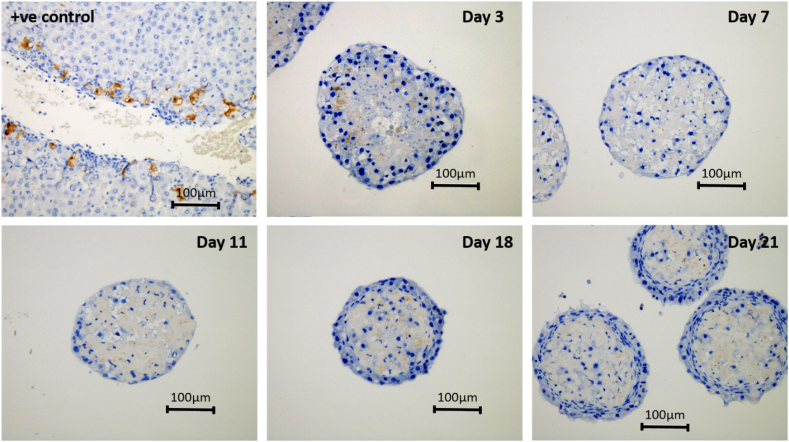
Cleaved-caspase 3 staining for analysis of apoptotic cell death. Spheroids were created on liquid-overlay plates from 5000 cells per well and fixed at day 3, 7, 11, 18 and 21 of culture, paraffin embedded, sectioned and stained with cleaved-caspase 3. Slides were counterstained with haematoxylin (blue) to stain for cell nuclei. Brown staining shows the presence of apoptosis. Images represent mid-sections through the spheroids at 20× magnification. Scale bars = 100 μm. (For interpretation of the references to colour in this figure legend, the reader is referred to the web version of this article.)

5000-cell spheroids where additionally stained over 21 days of the culture period with cleaved-caspase 3 which is an apoptotic cell marker (Fig. 8). This staining was carried out to assess the presence of cell death and as a means to support the findings from the histological analysis where we assert that PRH spheroids are devoid of the central necrotic core. Positive control slides were produced from rat liver sections. Cleaved-caspase 3 staining showed some areas of apoptosis at early culture time. However, later samples showed negative staining for apoptosis within the spheroids.

### 3.6 Ultrastructure analysis of hepatocytes

**Fig. 9:**
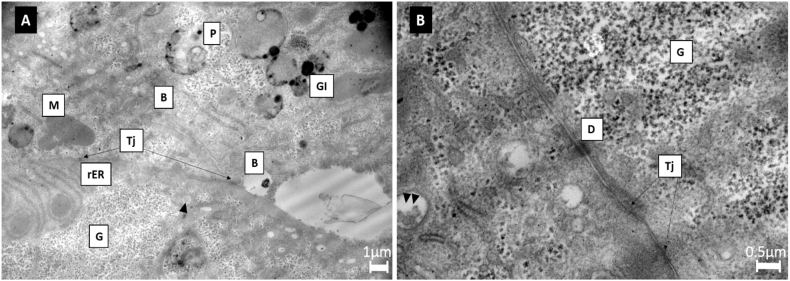
Ultrastructural feature of the spheroids by transmission electron microscopy (TEM). (A) shows a 5000-cell spheroid at day 3 of culture. TEM images showed characteristic hepatic ultrastructures. The presence of both glycogen vesicles (Gl) and granular glycogen (G) was abundantly expressed, as well as bile canaliculi (B) forming throughout the spheroid. The spheroids displayed a large quantity of metabolism-related organelles; including mitochondria (M), rough endoplasmic reticulum (rER), peroxisomes (P), and evidence of endocytosis or exocytosis (indicated by black arrow). There was also presence of tight junctions (Tj), indicating hepatic cell-cell interaction throughout the spheroid. (B) Ultrastructural features of the spheroids by transmission electron microscopy (TEM), day 7 of culture. TEM images displayed presence of glycogen (G), desmosomes (D) and specialised intracellular adherent junctions (Tj). Scale bar = 1 μm and 0.5 μm respectively.

Cellular ultrastructure was analysed utilising transition electron microscopy (TEM) analysis (Fig. 9). Upon spheroid formation (day 3), TEM images showed characteristic hepatic ultrastructures, and specialised intracellular adherent junctions could be seen clearly at day 7. Images were taken using a Gatan digital camera.

### 3.7 Internal structure of spheroids

**Fig. 10:**
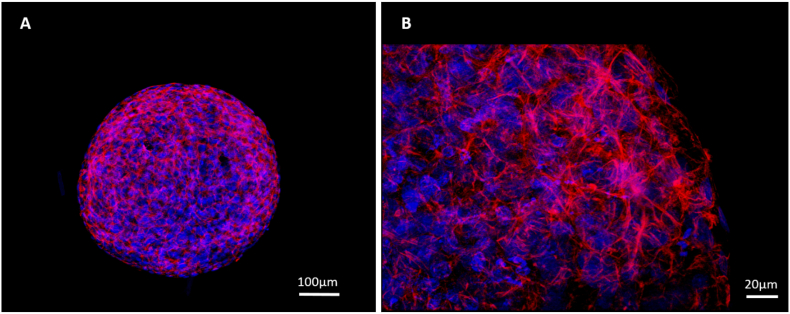
Formation of cytoskeletal F-actin throughout spheroids. 5000-cell Spheroids were cultured on liquid-overlay plates for 3 days (A) or 21 days (B), fixed and stained with phalloidin (red) to stain F-actin and Hoechst (blue) to stain the cell nuclei. A single plane snap shot was taken for (A) at 10× magnification whilst a maximum intensity projection image was taken for (B) at 40× magnification. Scale bars = 100 μm and 20 μm, respectively. (For interpretation of the references to colour in this figure legend, the reader is referred to the web version of this article.)

The internal structure of spheroids was analysed by IF analysis utilising phalloidin (red) to stain for F-actin and Hoechst (blue) as the standard stain for cell nuclei. Visualisation was performed by confocal microscopy (Fig. 10). Upon spheroid formation (day 3), F-actin filaments can be seen to form throughout the spheroid and at day 21, F-actin structures can be seen to remain throughout the spheroid body creating an intricate network throughout the entirety of the spheroid. F-actin staining demonstrates the cuboidal morphology of the cells within the spheroid.

### 3.8 Secondary structure formation in spheroids

**Fig. 11:**
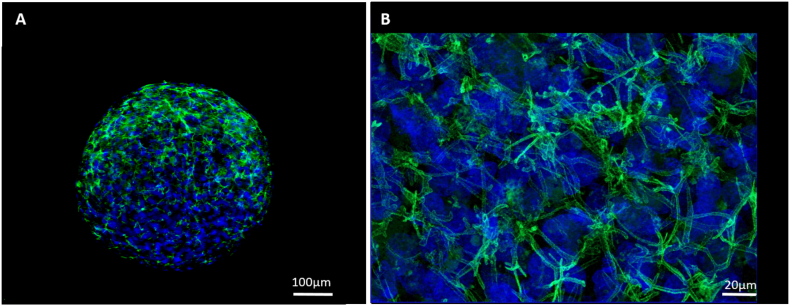
Bile canalicular formation throughout spheroids. 5000-cell Spheroids were cultured on liquid-overlay plates for 3 days (A) or 18 days (B), fixed and stained with P-gp (green) and Hoechst (blue) to stain the cell nuclei. A single plane snap shot was taken for (A) at 10× magnification whilst a maximum intensity projection image was taken for (B) at 40× magnification. Scale bars = 100 μm and 20 μm respectively. (For interpretation of the references to colour in this figure legend, the reader is referred to the web version of this article.)

Secondary structure formation (structural polarisation of hepatocytes) was determined *via* IF analysis. P-gp and MRP2 transporters are isolated to the apical/canalicular membrane of polarised hepatocytes. The expression of these two canalicular transporters was analysed to affirm the structural polarisation of the PRH within the spheroid and to confirm the formation of bile canalicular-like structures. Spheroids were analysed for up to 18 days for P-gp expression (Fig. 11). The staining pattern of the P-gp transporter was consistent with the staining seen with the phalloidin (Fig. 10). These secondary structures could be seen forming from day 3 of culture until at least day 18.

**Fig. 12:**
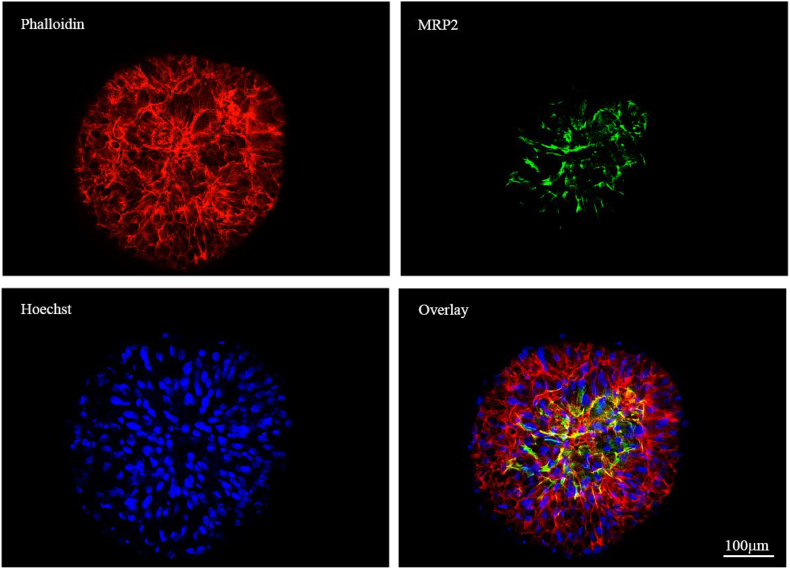
Bile canalicular formation throughout spheroids. Spheroids were cultured on liquid-overlay plates for 11 days, fixed and stained for MRP2 (green) and with Hoechst (blue) to stain the cell nuclei. 16 maximum intensity projection images were taken for Fig. 13 at 40× magnification using a Zeiss Axio Observer microscope and stitched together utilising a moving platform. Scale bars = 100 μm. (For interpretation of the references to colour in this figure legend, the reader is referred to the web version of this article.)

Spheroids were analysed for up to 11 days for MRP2 expression (see Fig. 12). Fig. 12 demonstrates the co-localisation of the canalicular transporter MRP2 (green) with the cytoskeletal F-actin (red) resulting in the yellow overlay image.

### 3.9 Analysis of transporter functionality

**Fig. 13:**
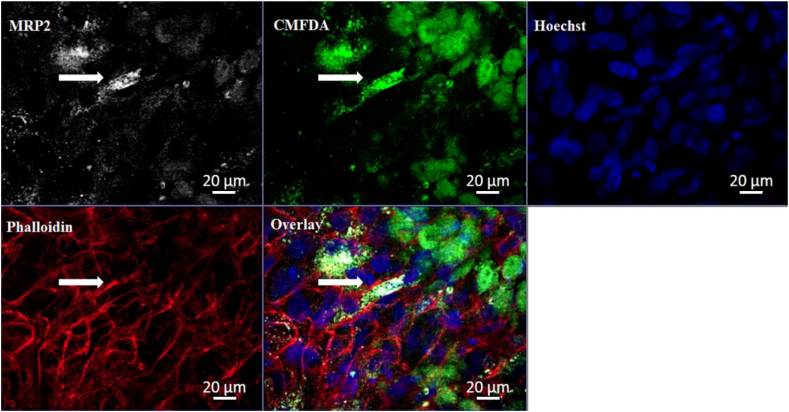
– Functional transport of CMFDA by MRP2 into secondary structures. Spheroids were cultured on liquid-overlay plates for up to 21 days. Spheroids were incubated with CMFDA (green) for 1 h, fixed and then stained with MRP2 (white), phalloidin (red) to stain F-actin and Hoechst (blue) to stain the cell nuclei. A maximum projection image was taken for at 40× magnification. White Arrows represent the overlay of the canalicular-like structures and substrate along with F-actin indicating co-localisation within the secondary structures. A Zeiss Axio Observer microscope was used for both images. Scale bars = 20 μm. (For interpretation of the references to colour in this figure legend, the reader is referred to the web version of this article.)

Functionality of secondary structures was assessed using fluorescently labelled dyes. Spheroids were incubated with 5 μM 5-chloromethylfluorescein diacetate (CMFDA-Thermo Fisher Scientific) for 1 h. Spheroids were washed in PBS and prepared for IF as previously described (see Fig. 13). PRH spheroids exhibited some limited retention of CMFDA substrates within the cell cytoplasm, but also and an accumulation and co-localisation of the CMFDA substrates within the secondary bile canalicular-like structures, shown by MRP2 staining (white) and complementary F-actin staining (red).

### 3.10 Urea quantification

**Fig. 14:**
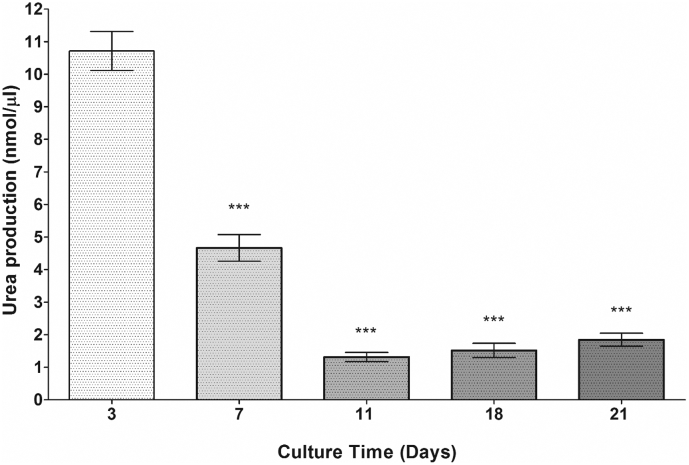
Urea synthesis analysis from 5000-cell spheroids. Spheroids were produced from on liquid-overlay plates. Urea secretion was quantified in 5000-cell spheroid supernatant samples over a 21 day culture period. Data are represented as mean ± SEM (*n* = 3 in triplicate). Urea synthesis was considered statistically significantly lower for all other time points compared to day 3 by One-way ANOVA, ^⁎⁎⁎^*p* < .001.

In order to assess hepatocyte functionality, both urea and albumin secretion was analysed as these end point analyses are demonstrative of liver-specific functionality. Urea synthesis from 5000-cell spheroids was quantified over 21 days of culture (Fig. 14). Urea synthesis decreased from 10.71 ± 0.60 nmol/μl to 1.32 ± 0.14 nmol/μl for 5000-cell spheroids from day 3 to day 11. Urea synthesis was at its lowest at day 11 for the 5000-cell spheroids but then increased over time to 1.85 ± 0.20 nmol/μl at day 21 of culture.

### 3.11 Albumin quantification

**Fig. 15:**
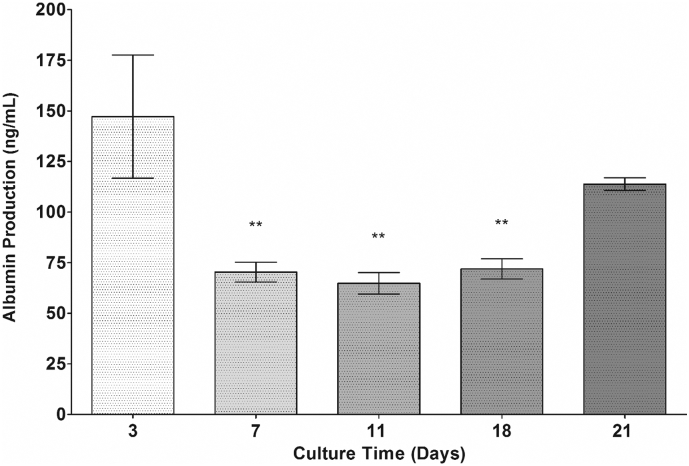
Albumin production from 5000-cell spheroids. Spheroids were produced from 5000 cells on liquid-overlay plates. Albumin production was quantified in supernatant samples over a 21 day period. Data are represented as mean ± SEM (n = 3 in triplicate). Albumin production was considered statistically significantly lower for day 7 and 11 time points compared to day 3 by One-way ANOVA, ^⁎⁎^*p* < .01. There was no statistically significant difference in albumin production between day 3 and day 21.

Albumin production from the 5000-cell spheroids was quantified over 21 days of culture period (Fig. 15). Albumin production initially decreased but stabilised and then increased over the culture period. Albumin production decreased from 147.20 ± 91.25 ng/ml to 64.80 ± 5.28 ng/ml from day 3 to day 11. From day 11 until day 21, albumin production steadily increased from the observed low of 64.80 ± 5.28 ng/ml to 113.80 ± 3.16 ng/ml.

## 4 Discussion

Previous research has shown 3D liver spheroids to be an improved *in vitro* model when compared with 2D cultures [15, 16, 27], so much so that there are now a number of companies that sell prepared spheroids as platforms for xenobiotic toxicity assessments (Messner et al., 2013). Hepatocyte spheroid models have been shown to maintain liver-specific functions for extended periods of time in culture (Bell et al., 2016; Chang and Hughes-Fulford, 2009). However, some of the key characteristics previously stated such as; size controllability, cellular polarisation and transporter functionality, needed to be evaluated in more detail. This more robust characterisation underlying the basic analysis of liver spheroid models represents an area that requires more attention within the field of *in vitro* liver models. In particular, structural and functional polarisation of hepatocytes within liver spheroid systems is an area that requires more in-depth investigation.

In this study, we have developed a cost-effective and reproducible methodology for producing viable PRH spheroids. PRH used in combination with the LOT had not previously been investigated. However, much interest had previously been given to the hanging-drop method, as implemented by Messner et al. *(*Messner et al., 2013*)*, and the utilisation of ready-made ULA plates (Bell et al., 2016). PRH were chosen because primary cells demonstrate a number of inherently advantageous characteristics when compared to the more frequently utilised hepatic-derived cell lines. Firstly, primary cells do not proliferate *ex vivo* and thus, regulation of the PRH spheroid size is more amenable compared with spheroids formed from a proliferative hepatic-derived cell line. It has previously been reported that C3A hepatocarcinoma cells display contact inhibited growth characteristics when compared with their parent HepG2 cells and as a result do not proliferate to the same extent when cultured in a spheroid conformation (Wrzesinski et al., 2013). However, histological and image analysis has demonstrated that the C3A cells within the spheroid body do proliferate over the duration of the culture time to the point where necrotic zones form (Gaskell et al., 2016). The formation of necrosis within 3D liver models over extended culture periods is one of the key difficulties that remains to date. The literature has previously reported that the physiologically occurring oxygen gradient was considered to be crucial for zonation within the liver lobule (Jungermann and Kietzmann, 2000). However, more recent investigations have reported that the oxygen gradient within the liver lobule is a regulator of zonation (Kietzmann, 2017). This is important when discussing the formation of necrotic regions within 3D liver models as this apparent lack of oxygen diffusion throughout the entirety of the spheroid body will inevitably negatively impact on zonated properties of the 3D model as well as gene expression and subsequent functionality. Within the liver lobule, blood coming from the portal triad flows through the sinusoids to the central vein. Due to the metabolism and elimination of xenobiotics, the composition of this blood changes and gradients of substrates and, in particular, oxygen are formed. As a result, there has been a notable drive in recent years to produce viable long-term 3D liver models that remain devoid of necrosis throughout the entirety of the culture period.

Phase contrast imagery demonstrated that over the course of our culture period the spheroid diameter decreases in size. The literature has reported this previously with other primary isolated spheroid cultures (Lin et al., 2006) and attributed the phenomenon to the up-regulation of key ECM components and cytoskeletal elements including E-cadherin and beta-1 integrin *etc.* Histological analysis validates the compact, cuboidal morphology of the hepatocytes that incorporate tight cell-cell interactions comparable with the morphology that hepatocytes exhibit *in vivo*. This is in contrast with conventional monolayer cultures whereby the cellular morphology is altered to a flattened state, and cells have relatively few cell-cell and cell-ECM interactions. This has previously been reported in HepG2 spheroids and monolayer cultures by Li et al. (Li et al., 2008). Our PRH spheroids show no visible sign of the characteristic central necrotic zones often observed in multicellular tumour spheroids which is partly due to the non-proliferative properties of primary hepatocytes *ex vivo*. Negative staining for cleaved-caspase-3 further strengthens the conclusions that our PRH model remains devoid of central cores of necrosis and cell death. Much of the literature shows that spheroids with a diameter > 150 μm form necrotic cores due to hypoxia and lack of nutrients (Funatsu et al., 2001). However, little quantitative research has focused on the factors and specific conditions that may contribute to the formation of these necrotic zones. These factors may include the cell type being cultured, the initial size of the spheroids upon formation, the specific oxygen consumption rate (OCR) of the cells and the culture conditions themselves. A much more robust analysis of these factors is required for the adequate characterisation and application of 3D liver microtissues.

IHC analysis was carried out to assess the peripheral cells constrained to the boundary of the spheroids. Vimentin staining has been shown to positively stain for mesothelial cells as well as fibroblasts, however, we conclude that the cells located at the periphery are likely to be mesothlial in nature due to their specific locality surrounding the spheroids and the findings from other previously reported research. Landry et al. (Landry et al., 1985) demonstrated the formation of the mesothelial lining similar to the Glisson membrane *via* positive staining with Vimentin, CK52 and CK55. The researchers did note, however, that in some cases spheroid linings composed of these stratified/elongated cells did not react with one of either markers. We see the same capsule-like structure forming for all seeding densities and at all time points supported by our IHC analysis, and therefore assert that this feature is the same as that described by Landry and colleagues. It is important to note that the formation of the capsule becomes extremely apparent by day 21. On day 18, we can see a thin cell layer lining the spheroid boundary, however, this rapidly thickens to a stratified outer membrane by day 21.

Specific transporters are expressed on the canalicular, and sinusoidal membranes of the hepatocytes (Esteller, 2008). Due to this transporter expression, bile canaliculi form between adjacent hepatocytes. The formation and maintenance of hepatocyte polarity is essential for a multitude of functions and we have been able to recapitulate this *in vivo-*like characteristic within our *in vitro* spheroid model. The establishment of functional polarisation was confirmed by carrying out IF analysis where canalicular membrane transporters (P-gp and MRP2) were stained and visualised. This analysis has previously been carried out with HepG2 and C3A spheroids (Gaskell et al., 2016). However, the bile canalicular networks present in the PRH spheroids are much finer, more extensive and complete when compared to the more globular-like secondary structures of HepG2 and C3A spheroids. As well as identifying the formation of these networks, we have been able to exhibit the functionality of these structures utilising CMFDA, a cell tracker that is passively taken up by hepatocytes. CMFDA substrates are actively transported out of the hepatocytes *via* MRP2. IF analysis revealed that this cell tracker substrate co-localises with the MRP2 transporter demonstrating functionality within our spheroids.

We were also able to demonstrate that PRH spheroids are able to stably produce albumin over the duration of the culture period as well as secreting urea. Urea secretion was seen to be significantly higher in PRH spheroids when compared to the previously characterised C3A and HepG2 spheroid (data not shown). Due to the isolation procedure of the primary rat hepatocytes, there are early decreases in both albumin and urea production. Over the duration of the culture period urea secretion remains low (but stable), potentially due to the cellular differentiation as demonstrated by IHC Vimentin staining. We suggest that the cell ratio of mesothelial cells to hepatocytes may account for the urea decrease over time. Conversely, albumin production increases over the duration of the culture period as the remaining fewer hepatocytes have greater (or equal) functionality when compared to the greater number of poorly functional hepatocytes at the early time point. Albumin production and urea secretion are well defined physiological functions of hepatocytes *in vivo* (Godoy et al., 2013) and therefore, the analysis of the production of these two functional end points provides an indicative measure of liver-specific functionality in our spheroid model. These findings help to assert that the culturing of cells in 3D provides a more representative model that recapitulates a number of *in vivo-*like characteristics that are not apparent in more commonly used monolayer cultures.

## 5 Conclusion

3D liver models have long been acknowledged as an improved platform for toxicological investigations due to the more representative morphology, improved functionality and increased viable culture periods provided when compared with 2D methods of culture. The technique described for the formation of PRH spheroids allows for a high-throughput platform with the potential for repeat-dose investigations. A key attribute of this spheroid system, particularly when compared with other spheroid models that utilise hepatic-derived cell lines, is the ability to be cultured over a 21-day period without the formation of necrotic regions. However, this is an area of research that requires further investigation with a view to characterise more precisely the causative factors for the formation of these necrotic regions, and if it is indeed simply the inability of oxygen to effectively diffuse >150 μm.

In conclusion, we have successfully developed a reproducible technique for creating uniform PRH spheroids and characterised their growth characteristics over a culture period of 21 days. The resultant spheroids exhibit an *in vivo*-like morphology, intricate cell-cell and cell-ECM interactions, as well as structural and functional polarisation. We additionally confirmed liver-specific functionality by analysing albumin production and urea secretion. We were also able to demonstrate that canalicular transporters, namely MRP2, were functional *via* transport of CMFDA substrates. Additionally, histological and IHC analysis of our PRH spheroid model has shown that the cells at the periphery of the spheroid seem to become distinctly different in morphology when compared with the more centralised hepatocytes. Only one other paper within the literature that we are aware of makes comment of the formation of a similar feature and identifies this to be the mesothelial lining of the liver *in vivo*, namely the Glisson's capsule (Landry et al., 1985). The formation of this *in vivo-*like feature confirmed *via* positive staining for Vimentin, further strengthens our assertion that this spheroid model is representative of the *in vitro* liver. The culmination of these analyses demonstrates that our PRH liver spheroid model recapitulates the *in vivo* liver more closely than currently utilised 2D cultures, and may be well suited for repeat-dose hepatotoxicity investigations.
